# Activation of a cGAS-STING-mediated immune response predicts response to neoadjuvant chemotherapy in early breast cancer

**DOI:** 10.1038/s41416-021-01599-0

**Published:** 2021-11-02

**Authors:** Eileen E. Parkes, Kienan I. Savage, Tong Lioe, Clinton Boyd, Sophia Halliday, Steven M. Walker, Keith Lowry, Laura Knight, Niamh E. Buckley, Andrena Grogan, Gemma E. Logan, Alison Clayton, Jane Hurwitz, Stephen J. Kirk, Jiamei Xu, Fatima Abdullahi Sidi, Matthew P. Humphries, Victoria Bingham, Neo-DDIR Investigators, Melvyn Ang, Conal Askin, Louise Bamford, Ruth Boyd, Miriam Buckley, Jacqueline Clarke, Lynn Darragh, Elaine Davis, Jennifer Foreman, Rebecca Gallagher, Janine Gill, Michael Hanna, Naomi Hill, Gareth Irwin, Peter Mallon, Seamus McAleer, Joanne McAllister, Melanie Morris, Nicole Pierce, Sigi Refsum, Samantha Sloan, Sinead Treanor, Jaqueline A. James, Colin R. James, D. Paul Harkin, Richard D. Kennedy, Stuart A. McIntosh

**Affiliations:** 1 Patrick G Johnston Centre for Cancer Research, Queen’s University Belfast, 97 Lisburn Road, Belfast, BT9 7AE UK; 2 Belfast Health and Social Care Trust, Belfast City Hospital, Lisburn Road, Belfast, BT9 7AB UK; 3 Department of Oncology, Medical Sciences Division, University of Oxford, Oxford, UK; 4 Almac Diagnostic Services, Almac Group, 19 Seagoe Industrial Estate, Craigavon, BT63 5QD UK; 5 School of Pharmacy, Queen’s University Belfast, 97 Lisburn Road, Belfast, BT9 7AE UK; 6 South Eastern Health and Social Care Trust, Ulster Hospital, Upper Newtownards Road, BT 16 1RH Dundonald, UK; 7 Precision Medicine Centre, Patrick G Johnston Centre for Cancer Research, Queen’s University Belfast, 97 Lisburn Road, Belfast, BT9 7AE UK

**Keywords:** Breast cancer, Predictive markers

## Abstract

**Background:**

The DNA-damage immune-response (DDIR) signature is an immune-driven gene expression signature retrospectively validated as predicting response to anthracycline-based therapy. This feasibility study prospectively evaluates the use of this assay to predict neoadjuvant chemotherapy response in early breast cancer.

**Methods:**

This feasibility study assessed the integration of a novel biomarker into clinical workflows. Tumour samples were collected from patients receiving standard of care neoadjuvant chemotherapy (FEC + /−taxane and anti-HER2 therapy as appropriate) at baseline, mid- and post-chemotherapy. Baseline DDIR signature scores were correlated with pathological treatment response. RNA sequencing was used to assess chemotherapy/response-related changes in biologically linked gene signatures.

**Results:**

DDIR signature reports were available within 14 days for 97.8% of 46 patients (13 TNBC, 16 HER2 + ve, 27 ER + HER2-ve). Positive scores predicted response to treatment (odds ratio 4.67 for RCB 0-1 disease (95% CI 1.13–15.09, *P* = 0.032)). DDIR positivity correlated with immune infiltration and upregulated immune-checkpoint gene expression.

**Conclusions:**

This study validates the DDIR signature as predictive of response to neoadjuvant chemotherapy which can be integrated into clinical workflows, potentially identifying a subgroup with high sensitivity to anthracycline chemotherapy. Transcriptomic data suggest induction with anthracycline-containing regimens in immune restricted, “cold” tumours may be effective for immune priming.

**Trial registration:**

Not applicable (non-interventional study). CRUK Internal Database Number 14232.

## Introduction

Breast cancer is a heterogeneous disease, encompassing multiple molecular subtypes, some of which have targeted therapies available, including endocrine therapy and anti-HER2 therapies for oestrogen receptor-positive (ER+) and human epidermal growth factor 2 positive (HER2+) disease, respectively. Despite this, patients receiving chemotherapy are most frequently treated with anthracycline-taxane containing regimens, regardless of subtype. In the neoadjuvant setting, ~18% of unselected patients will have a pathological complete response (pCR) to treatment, although there is currently no reliable method of predicting this [1]. Importantly, the highest response rates to DNA-damaging agents, including anthracyclines, are seen in patients with DNA-repair-deficient tumours. For example, *BRCA1/2*-mutant tumours are more sensitive to DNA-damaging agents, including anthracyclines and carboplatin [2, 3]. However, anthracyclines have important long-term toxicities, particularly cardiotoxicity, when compared with non-anthracycline regimens [4, 5]. Furthermore, the addition of trastuzumab to anthracycline-containing regimens has been shown to substantially increase the incidence of cardiotoxicity [6]. Finally, anthracycline-containing regimens are associated with a small excess in cases of myelodysplasia and acute myeloid leukaemia [7]. Given these toxicities, there is clearly a pressing clinical need to identify patients likely to benefit from DNA-damaging agents prior to treatment.

Recently, we identified an immune-driven 44-gene signature recognising the loss of the Fanconi Anemia (FA)/BRCA DNA-repair pathway— the DNA Damage Immune Response (DDIR) signature [8]. The assay was developed using DNA microarray data from patients with FA and from breast tumours (enriched for BRCA1/2-mutant-associated tumours). Unsupervised hierarchical clustering allowed the identification of a subgroup of cases with an innate DNA-repair deficiency, characterised by upregulation of a subset of genes associated with immune signalling. In the retrospective analysis, this 44-gene signature was developed and validated across all breast cancer molecular subtypes to predict response to DNA-damaging chemotherapy in both the adjuvant and neoadjuvant treatment of breast cancer, predicting 5-year relapse-free survival post-adjuvant treatment in DDIR-positive patients, with a hazard ratio of 0.37 (95% CI 0.15–0.88, *P* = 0.03). Moreover, the signature has been shown to be prognostic in triple-negative breast cancer (TNBC), with DDIR positivity associated with improved disease-free and overall survival in patients treated with adjuvant doxorubicin/cyclophosphamide chemotherapy [9].

The DDIR-positive molecular subtype is characterised by the upregulation of immune genes, including cytokines and immune-checkpoint genes [8]. Using preclinical models, we demonstrated constitutive activation of the cGAS-STING pathway in DNA-repair-deficient tumours to be the underlying mechanism for this innate immune response [10]. In keeping with this, breast tumours with a high DDIR signature score are associated with increased tumour-infiltrating lymphocytes (TILs) at diagnosis, including CD4^+^ and CD8^+^ T lymphocytes [9, 10]. Activation of the cGAS/STING pathway has been linked with the upregulation of immune-checkpoint genes, which was also observed in DDIR-positive breast tumours [8, 10]. Taken together, this suggests that DDIR-positive tumours are characterised by an inflamed yet immune restricted tumour microenvironment, where increased immune infiltrates coexist with upregulated immune checkpointing genes. In breast cancer, activation of an anti-tumour immune response and the presence of TILs is linked with both increased pathological complete response (pCR) rates and improved long-term outcomes after neoadjuvant chemotherapy [11, 12]. Consequently, the DDIR signature may identify a group of tumours characterised by immune activation that respond to DNA-damaging chemotherapy, potentially providing a predictive biomarker with clinical utility in the neoadjuvant setting in early breast cancer.

The primary objective of this study was to prospectively determine the feasibility of integrating the DDIR signature into clinical workflows, with a report obtained within 14 days (judged to be a realistic timeframe for the use of this novel, previously validated biomarker in clinical decision-making). Secondary endpoints included the ability of the assay to predict response to standard neoadjuvant chemotherapy (NACT) in the treatment of early breast cancer. Gene expression profiling and histological analysis of pre- and post-chemotherapy biopsies were performed to identify immune responses and other potentially targetable biologies in breast tumours treated with NACT, as exploratory endpoints.

## Methods

### Patients and methods

Patients with a histologically confirmed diagnosis of breast cancer suitable for neoadjuvant treatment were recruited at two Northern Ireland centres (Belfast City Hospital and Ulster Hospital, Dundonald) between April 2014 and August 2017. Women who were 18 years or older, WHO performance status 0–1, with T1-3, N0-2 (AJCC 8th Edition) or inflammatory breast cancer were eligible. Inclusion/exclusion criteria are outlined in Supplementary Fig. 1. The study was approved by the Office for Research Ethics Committee Northern Ireland (Reference: 13/NI/0107).

Tumour grade was identified on diagnostic biopsies. Biomarker status (oestrogen receptor (ER), progesterone receptor (PR) and HER2) was determined centrally; methods are described in Supplementary Materials. Nodal status was assessed at diagnosis by axillary ultrasound + /− fine needle aspiration cytology. Pre-treatment sentinel lymph node biopsy (SLNB) was carried out if axillary staging was negative; patients with nodal involvement determined either by cytology or SLNB underwent axillary lymph node dissection following neoadjuvant therapy at the time of definitive breast surgery.

As the primary endpoint of the study was the feasibility of returning an assay report within 14 days, an arbitrary sample size of 50 patients was chosen; there was no formal power calculation for secondary or exploratory endpoints. Details of statistical analysis are provided in the Supplementary Materials.

### Interventions

The trial schema is outlined in Supplementary Fig. 1A. Following informed consent, two additional 14-G needle tumour biopsies, one formalin-fixed paraffin-embedded (FFPE) and one fresh frozen (FF), were obtained. Patients were treated according to local guidelines. All patients received three cycles of FEC “100” (5-fluorouracil 500 mg/m^2^, epirubicin 100 mg/m^2^ and cyclophosphamide 500 mg/m^2^ given intravenously q3 weeks) chemotherapy and were then treated with three further cycles of FEC “100” or taxane treatment with the addition of trastuzumab/pertuzumab concurrently with taxanes in HER2-positive tumours. No patients in this study received platinum salts as this was not standard of care in these institutions at the time of this study. Further FFPE biopsies/resection specimens were taken at the time points indicated in Supplementary Fig. 1A. DDIR scores were not reported to either treating clinicians or patients and the report was not used to select treatment; patients received standard of care neoadjuvant therapy, with the choice of regimen at the discretion of their treating physician. Definitive breast surgery was carried out within 4–6 weeks after the completion of systemic therapy.

### Gene expression profiling

H&E sections were annotated for tumour content. A minimum estimated tumour content of 80% viable tumour nuclei within the annotated area was mandated. Following annotation, 10 × 6-µm sections were cut and annotated tumour macrodissected for RNA extraction. cDNA microarray profiling was performed using the Almac Breast DSA (Almac Diagnostic Services, Craigavon, UK) as previously described, as this is the technology on which the signature has been validated for clinical use [3]. For exploratory analysis, to aid understanding of the underpinning biology, RNA sequencing was performed on RNA extracted from pre- and post-treatment FFPE specimens, using the Illumina Tru-Seq^®^ RNA Exome library preparation kit, followed by sequencing on the Illumina NextSeq with paired-end reads (75 bp) and 50 M reads per sample. Details of alignment and expression calculation are provided in Supplementary Material.

### Determination of DDIR score

Microarray data were pre-processed using the Robust Multi-array Average (RMA) method [13], as previously described [8]. The median expression of each of the DDIR genes was determined and DDIR score was calculated based on the weighted sum of these genes. A previously published, pre-defined threshold of 0.3681 was used to define DDIR signature status, whereby a score ≥0.3681 was classified as DDIR positive and <0.3681 classified as DDIR negative [8].

### Assessment of pathological response

Surgical resection was performed within 4–6 weeks of the final cycle of NACT. Pathological response rates were measured on resection specimens using Residual Cancer Burden (RCB) scores [14]. RCB was assessed as 0, 1, 2 or 3 where 0 refers to pathological complete response (AJCC ypT0/ypTis ypN0) and 3 to extensive residual disease. RCB scoring was reported as this is a validated measure of response to therapy and is recommended for use in neoadjuvant chemotherapy trials [15]. Pathologists were blinded to the DDIR score.

### Identification and quantification of TILs

Tumour-infiltrating lymphocytes (TILs) were defined as the percentage of stromal TILs within the margins of the tumour. These were scored as a continuous percentage and reported in accordance with the recommendations of the International Immuno-Oncology Working Group on Breast Cancer for both baseline and residual tumour specimens. [16, 17]. TILs were scored by two independent observers on 4-μm haematoxylin and eosin (H&E) sections of biopsy samples taken at diagnosis, after three cycles of NACT and at surgical resection. Observers were blinded to DDIR and RCB scores. Good concordance was identified between observers with a 78.48% agreement (Cohen’s kappa co-efficient = 0.536).

### clara^T^ total mRNA report

The raw sequence data in FASTQ format were normalised to FPKM data matrices and processed through Almac’s proprietary analysis pipeline and reporting software for generation of the Version 2.0.0 clara^T^ Total mRNA Report (Almac Diagnostic Services, https://www.almacgroup.com/diagnostics/claratreport/). clara^T^ is a software-driven solution, classifying biologically relevant gene expression signatures into several text and graphical reports. Version 2.0.0 clara^T^ Total mRNA Report, reports on six key biologies (Immuno-Oncology (IO), Epithelial to Mesenchymal Transition (EMT), Angiogenesis, Proliferation, Cell Death & Genome Instability) by providing expression of 62 unique gene expression signatures, 60 single gene drug targets and 3952 single genes relevant to the six biologies for exploratory analysis. Further detail on clara^T^ is provided in the Supplementary Material.

### Multiplex immunofluorescence staining

Sequential 3-µm sections were obtained from FFPE tumour blocks. Serial sections were stained with two previously validated multiplex panels conducted using Opal 7-Color Automation IHC Kit (Akoya Biosciences, Marlborough, MA, USA) [18]. Details of the panels are provided in Supplementary Materials. Automated staining was conducted on a Leica Bond Rx. Optimised retrieval methods and sequential staining steps for Opal are detailed in Supplementary Table 1 and were used according to the manufacturer’s instructions. Image analysis details are provided in Supplementary Materials.

## Results

### Trial enrolment and DDIR scores

**Fig. 1:**
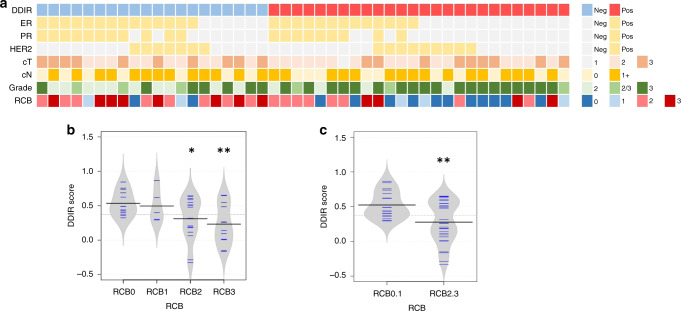
Tumour characteristics and response to NACT according to DDIR status. **a** Tumour characteristics: each column represents one patient. DDIR status is shown on the top row with the associated key. Hormone and HER2 receptor status are shown for each tumour, clinical T and N stages and grade. RCB = residual cancer burden score where 0 = complete pathological response, 1 = minimal residual disease, 2 = moderate residual disease and 3 = extensive residual disease. No significant difference in patient characteristics was identified except for grade, where DDIR-positive tumours were noted to be higher grade than those classified as DDIR negative (*P* = 0.024, unpaired *t* test, data in Table 1). **b** DDIR score and RCB (response) to NACT. Tumours that demonstrated RCB 0–1 clinical response had significantly higher DDIR scores than those with RCB 2–3 responses (*P = 0.0286, **P = 0.0042, unpaired *t* test, 46 cases). **c** Compared collectively, tumours with a clinical response of RCB 0–1 to NACT had significantly higher DDIR assay scores than those with RCB 2–3 responses. (**P = 0.0033, unpaired *t* test, 46 cases).

Trial enrolment is summarised in Supplementary Fig. 1B. Fifty-two patients were recruited, with one withdrawal and two patients excluded (shown in the CONSORT flow diagram). Three diagnostic biopsy samples (6.1%) contained insufficient tumour content for RNA extraction, leaving 46 patients for analysis. DDIR signature results were available within 14 days for 45 patients (97.8%). Figure 1a shows patient and tumour characteristics at an individual patient level. Table 1 summarises patient/tumour pathological characteristics, treatment regimens given and pathological response to treatment, according to DDIR status. Twenty-six patients had DDIR-positive tumours, and 20 DDIR negative. Tumour grade was higher in DDIR-positive tumours (on baseline assay score) compared to DDIR-negative (*P* = 0.02). Other clinicopathological characteristics, including ER/PR and HER2 status, as well as lymph node involvement, were similar between DDIR-positive and negative tumours (*P* values in Table 1).

**Table 1:** Clinicopathological characteristics of study patients.

	All patients	DDIR-positive	DDIR-negative	
	(*n* = 46)	(*n* = 26)	(*n* = 20)	
Age at diagnosis (years)	28–64	35–64	28–58	*P* = 0.813^a^
	(median = 47)	(median = 47)	(median = 48)	
ER-positive HER2-negative	27 (59%)	13 (48%)	14 (52%)	*P* = 0.41^b^
HER2-positive (*n*)	16 (35%)	9 (56%)	7 (44%)	
ER+ HER2+	11 (24%)	5 (45%)	6 (55%)	
ER− HER2+	5 (11%)	4 (80%)	1 (20%)	
Triple-negative	13 (28%)	8 (62%)	5 (38%)	
T stage				*P* = 0.37^c^
T2	27	17 (63%)	10 (37%)	
T3	19	9 (47%)	10 (53%)	
N				*P* = 0.23^c^
Neg	12	5 (42%)	7 (58%)	
Pos	34	21 (62%)	13 (38%)	
Grade				*P* = 0.02^b^*
2	14	4 (29%)	10 (71%)	
2/3	6	3 (50%)	3 (50%)	
3	26	19 (73%)	7 (27%)	
Treatment regimen				*P* = 0.35^c^
FEC 100	8	3 (37%)	5 (63%)	
FEC-taxane	38	23 (61%)	15 (39%)	
Neoadjuvant anti-HER2-targeted treatment	13	5 (38%)	8 (62%)	
Pre-treatment SLNB	16	8	8	*P* = 0.57^c^
SLNB +ve	4	3 (75%)	1 (25%)	
SLNB −ve	12	5 (42%)	7 (58%)	
RCB class				*P* = 0.03^c^*
RCB0-1	18	14 (78%)	4 (22%)	
RCB2-3	28	12 (43%)	16 (57%)	

^a^Mann–Whitney test.

^b^Chi-squared test (*P < 0.05).

^c^Fisher’s exact test (*P < 0.05).

### DDIR assay predicts response to NACT

Patients with DDIR-positive tumours had an odds ratio of 4.667 for either complete pathological response to NACT, or minimal residual disease (MRD) (RCB 0–1) (95% CI 1.131–15.09, *P* = 0.0324). Only 4 out of 20 patients with DDIR-negative tumours had a pathological response to treatment (RCB 0–1), in comparison to 14 out of 26 DDIR-positive tumours. Tumours with RCB 2 or 3 (moderate or extensive residual disease post NACT) had significantly lower DDIR scores (median 0.2607) than RCB 0–1 (median 0.4636) compared individually (*P* = 0.0286 and 0.0042, respectively) or collectively (*P* = 0.0033) (Fig. 1b, c). Similarly, tumours with a pathological complete response (pCR, RCB 0) to NACT had significantly higher DDIR scores than those with any residual disease (RCB 1–3) (*P* = 0.0152) (Supplementary Fig. 2). Indeed, DDIR-positive tumours had an odds ratio of 6.60 (95% CI 1.334–32.27, *P* = 0.0217) for complete pathological response (RCB 0) versus not (RCB 1-3). In multivariate analysis (Supplementary Table 2), DDIR status remained an independent predictor of response, along with HER2 status.

### Tumour-infiltrating lymphocytes

**Fig. 2:**
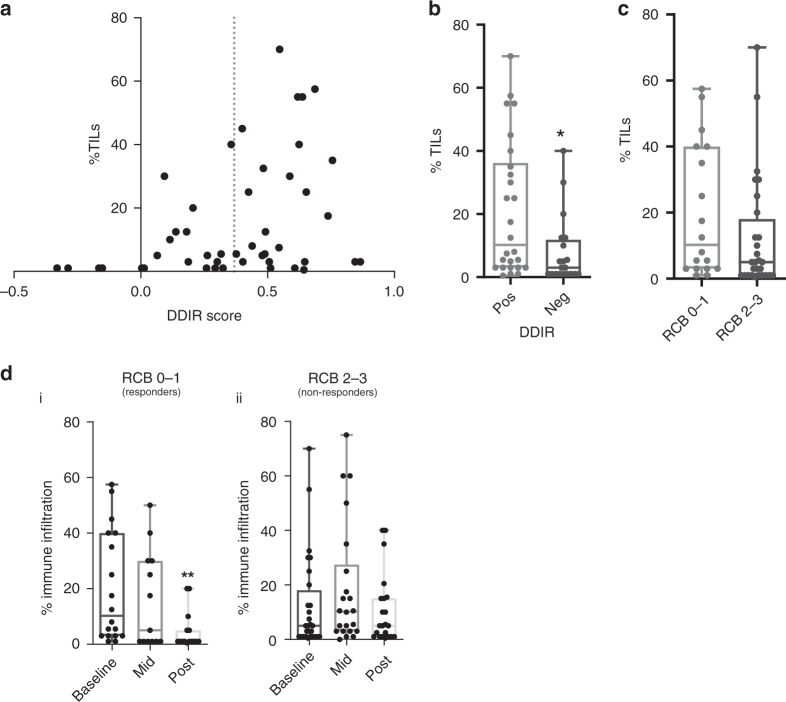
Tumour-infiltrating lymphocytes and response to NACT. **a** Correlation between DDIR score and tumour-infiltrating lymphocytes (TILs). An increasing percentage of stromal TILs correlates with increasing DDIR score. Dotted line shows threshold for DDIR negative/positive cut-off (*r* = 0.3386, *P* = 0.0213, Spearman correlation, 46 evaluable cases). **b** DDIR-positive tumours had significantly increased lymphocytic infiltration at baseline compared to tumours classified as DDIR negative. (*P = 0.0144, unpaired *t* test, 46 evaluable cases). **c** Percentage stromal TILs at baseline according to response to NACT using RCB score. No significant difference in TILs was identified in tumours with RCB 0 or 1 (median 10%) compared to RCB 2 or 3 (median 5%) responders. (*P* = 0.123, unpaired *t* test, 46 evaluable cases). **d** (i) Response to NACT was associated with reduced TILs at resection compared to baseline. (*P* = 0.0072, unpaired *t* test, 15 paired samples). (ii) No significant difference in TILs between baseline and resection was seen in non-responding tumours, with a non-significant increase noted following three cycles of FEC. (*P* = 0.0792, unpaired *t* test, 22 paired samples).

As previously reported [10], increasing DDIR score correlated with increased TILs (*P* = 0.0213, Spearman’s correlation, *r* = 0.3386) (Fig. 2a). DDIR-positive tumours had significantly higher TIL levels at baseline than DDIR-negative tumours (median TILs 20% vs 7.5%; *P* = 0.0144) (Fig. 2b). However, comparison of baseline TILs in responders to NACT (RCB 0–1) compared to tumours with moderate/extensive residual disease (RCB 2-3) did not reveal a significant difference (Fig. 2c) (*P* = 0.179). Similarly, no significant difference was observed between TIL levels at baseline in patients with a pCR, compared to those with any residual disease (*P* = 0.3453) (Supplementary Fig. 3A). In a multivariate analysis including tumour grade, ER and HER2 status, DDIR status and TIL levels, TILs were not seen to independently predict response to treatment (Supplementary Table 2).

TIL levels were not significantly different between breast cancer subtypes (Supplementary Fig. 3B). Changes in DDIR score before and after three cycles of FEC-100 are shown for DDIR-negative (Supplementary Fig. 4A) and DDIR-positive tumours (Supplementary Fig. 4B), showing, respectively, a non-significant rise and fall in DDIR scores with treatment. Changes in TIL level during treatment according to DDIR score status are shown in Supplementary Fig. 5A and B with a significant reduction in TILS seen in DDIR-positive tumours between baseline and surgery (*P* = 0.0254). Supplementary Fig. 5C and D illustrates the changes in TIL levels in DDIR-positive responding and non-responding tumours, demonstrating significant reductions in TILs in DDIR patients with a good response to treatment (*P* = 0.007). A non-significant increase in TIL levels was seen in DDIR-negative tumours following chemotherapy.

Strikingly, in patients with pCR or MRD (RCB 0–1), TILs were absent or markedly reduced at the completion of NACT in resection specimens (*P* = 0.0072) (Fig. 2d). In comparison, in non-responding tumours, TILs showed a non-significant rise following three cycles of FEC chemotherapy (Fig. 2d), in keeping with the observation that anthracyclines are immune stimulants [19–21]. Non-responding tumours were noted to have persistent lymphocytic infiltrate at resection (Fig. 2d).

### Immune cell populations and signalling in DDIR-negative and -positive tumours

**Fig. 3:**
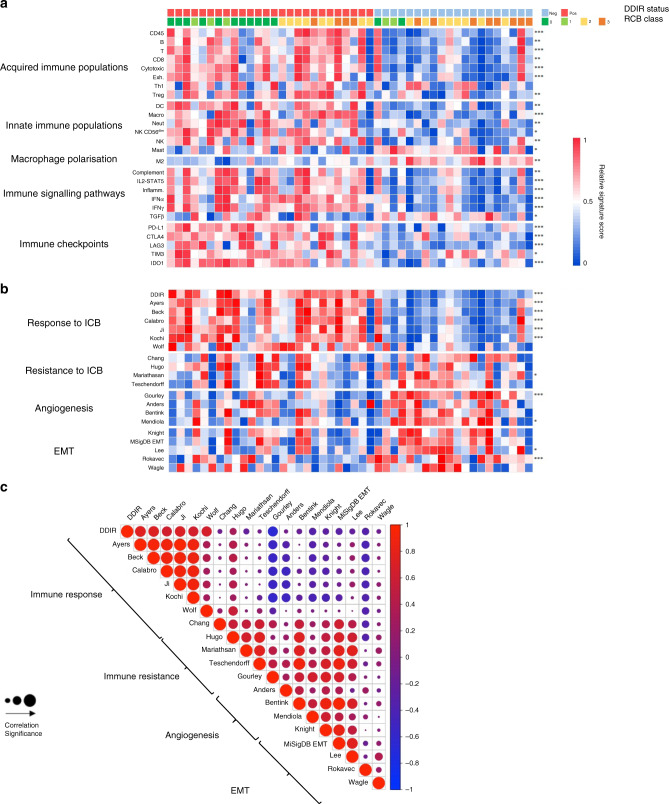
Immune signalling, angiogenesis and EMT in DDIR-negative and -positive tumours. **a** Panels 1 and 2 = Gene signatures predicting acquired (panel 1) and innate (panel 2) immune cell infiltrating populations in DDIR-negative (left) and DDIR-positive (right) tumours; ex T cells   exhausted T cells, DC   dendritic cells, NK   natural killer cells. Panel 3 = macrophage polarisation. Panel 4 = Immune signalling pathways identified as active in DDIR assay-positive and -negative tumours. Panel 5 = Expression of immune-checkpoint genes in DDIR-negative and positive tumours. DDIR status and RCB score (0–3) is indicated on *x* axis for all 46 samples. *P < 0.05; **P < 0.01; ***P < 0.001, unpaired *t* test. **b** Panel 1 = Gene signatures derived from clara^T^ analysis and reported to predict response to immune-checkpoint blockade, as well as the DDIR assay [8, 28–30, 58–60]. Panel 2 = Gene signatures derived from claraT analysis and reported to predict resistance to ICB in DDIR-negative and -positive tumours [27, 31–33]. Panel 3 = Gene signatures associated with increased angiogenic signalling or response to anti-angiogenic agents [34–36, 61]. Panel 4 = Gene signatures associated with increased EMT signalling [26, 37–39, 62]. DDIR status and RC score (0–3) is indicated on *x* axis for all 46 samples. *P < 0.05; **P < 0.01; ***P < 0.001, unpaired *t* test. **c** Correlations of gene signatures predicting angiogenic, EMT or immune signalling. Signatures predicting angiogenic or EMT signalling negatively correlated with those predicting immune signalling. Significance of correlation is shown using size, red indicates a positive correlation and blue negative correlation (gene signature references as for Fig. 4b).

**Fig. 4:**
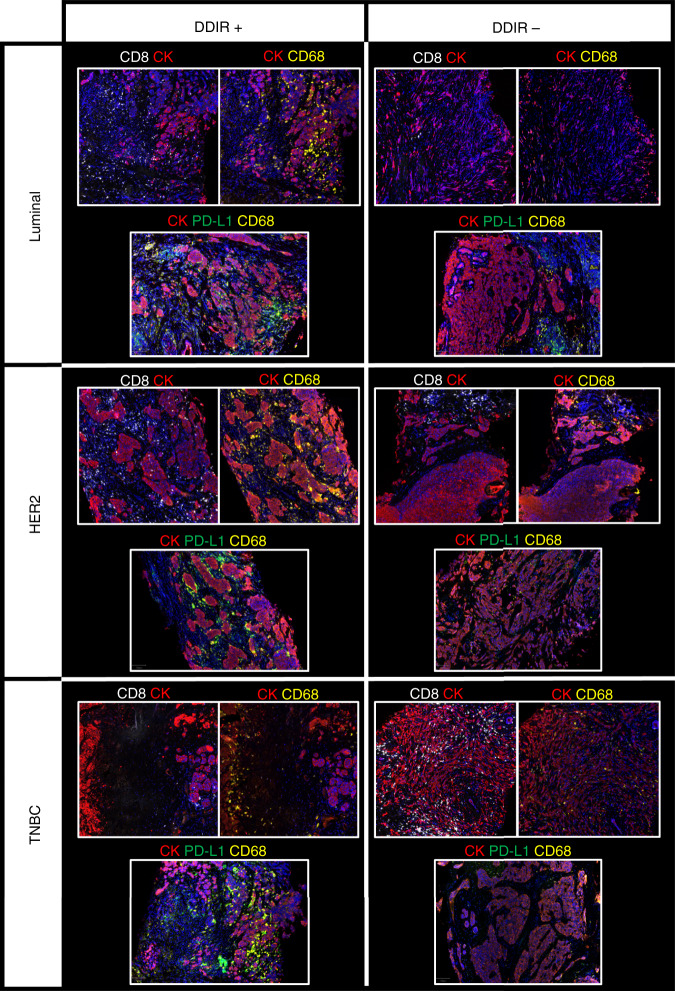
T-cell/macrophage expression and PD-L1/macrophage expression across breast cancer subtypes, by DDIR status. From top to bottom, luminal, HER2-positive and triple-negative breast cancer (TNBC) cases are illustrated and have been stained for cytokeratin (red), CD68 macrophages (yellow), CD8 T cells (white) and PD-L1 immune-checkpoint protein (green) expression. DDIR-positive cases are shown on the left and DDIR-negative cases on the right, illustrating higher CD68 and CD8 expression in DDIR-positive tumours, and co-localisation of PD-L1 and CD68 in DDIR-positive cases. All images are shown at ×4 magnification.

Transcriptomic data from baseline tumour biopsies were used to identify immune infiltrating populations in both DDIR-negative and -positive tumours, using previously described, well-validated gene signatures [22, 23]. Signature scores representing adaptive immune-response cells (B and T lymphocytes) were significantly higher in DDIR-positive compared to DDIR-negative tumours (Fig. 3a). Notably, signatures representative of exhausted T cells were also higher in DDIR-positive breast tumours (*P* < 0.001). We then identified innate immune populations in DDIR-negative and -positive tumours (Fig. 3a). While again most signature scores representing innate immune cell populations were significantly higher in DDIR-positive cancers compared to DDIR-negative cancers pre-treatment, mast cell signature scores were lower in DDIR-positive tumours (*P* < 0.05). Importantly, mast cells have been associated with increased angiogenesis in a number of solid tumours [24]. In addition, signature scores representing macrophages were higher in DDIR-positive tumours (*P* < 0.001), and this finding was confirmed across breast cancer subtypes with immunohistochemical staining for CD68 (Fig. 4). Furthermore, despite overall levels lower of CD68 expression, the low levels of CD8 expression also seen in DDIR-negative tumours resulted in higher CD68/CD8 gene expression ratios, implying these tumours had a higher proportion of “M2” polarised macrophages (which have been associated with an immunosuppressive tumour microenvironment (TME) [25], compared to DDIR-positive tumours (Fig. 3a). This finding was validated at the protein level using multiplex fluorescence immunohistochemical staining for CD8^+^ and CD68^+^ infiltration in tumour core biopsies, (Fig. 4). As expected, CD8 positivity was significantly higher in DDIR-positive than negative tumours (median CD8 positivity 12.3% vs 6.24%, *P* = 0.037), as was CD68 positivity (median 4.33% vs 0.955%, *P* = 0.029) (Supplementary Fig. 6).

We also utilised the molecular signatures database (MSigDB) scoring system [26] to assess immune hallmarks within tumours. This identified upregulation of interferon-alpha and gamma, complement and IL2-STAT5 signalling pathways in DDIR-positive tumours (*P* < 0.001) (Fig. 3a). Interestingly, a significant increase in transforming growth factor-beta (TGF-β) signalling (*P* < 0.05) was identified in DDIR negative tumours, in keeping with immune exclusion in this subgroup of breast tumours [27].

### Immune-checkpoint gene expression is upregulated in DDIR-positive tumours

The immune-checkpoint genes PD-L1 (*CD247*)*, CTLA4, LAG3*, TIM3 (*HAVCR2*) and *IDO1* were assessed using gene expression data, with significantly higher expression of each in DDIR-positive tumours (Fig. 3a). Multiplex immunofluorescence (IF) for PD-L1 and CD68 in DDIR-positive tumours confirmed that the majority of the PD-L1 expression co-localised with macrophages (Fig. 4). In the transcriptomic data, normalised PD-L1 expression has been shown to correlate with the Prat macrophage-infiltration signature (Supplementary Fig. 7A, Spearman correlation, *r* = 0.7869, *P* < 0.0001). This was confirmed at the protein level, using the multiplex IF data to correlate CD68 and PD-L1 expression (Supplementary Fig. 7B, Spearman correlation, *r* = 0.67, *P* < 0.001). Together with the data above, this suggests that DDIR-positive tumours maintain tumour growth in the presence of immune infiltration via upregulation of immune checkpoints, with the macrophage population a key contributor to the upregulation of PD-L1.

We assessed a further six gene signatures [28–30] associated with immune infiltration and potential response to immune-checkpoint blockade (ICB). All signature scores were significantly higher in DDIR-positive tumours (Fig. 3b). However, gene signatures associated with predicted resistance to ICB and fibroblast response [31–33] did not show a significant difference between DDIR-negative and -positive tumours (Fig. 3b).

In summary, DDIR-positive tumours showed increased immune infiltration with high immune-checkpoint gene expression. As expected, PD-L1 and *IDO1* expression were elevated in DDIR-positive tumours, given that these genes are constituent parts of the signature; however, upregulation of non-signature checkpoint genes was also seen in the transcriptomic data. In contrast, DDIR-negative tumours have a paucity of immune cell infiltration, with increased TGF-β signalling.

### Identification of potentially targetable biologies in DDIR-negative tumours

In addition, we identified upregulation of angiogenic signalling in DDIR-negative tumours, which is in keeping with the high mast cell signature scores observed in these tumours [34–36] (Fig. 3b). In addition, as TGF-β signalling was increased in DDIR-negative tumours, we asked if there was concomitant upregulation of epithelial–mesenchymal transition (EMT). Indeed, we observed higher EMT signalling in DDIR negative, relative to DDIR-positive tumours [26, 37–39] (Fig. 3b).

While each signature correlated strongly with others predicting similar biology, there was a marked inverse correlation between signatures associated with increased angiogenesis and EMT signalling and those associated with immune response (Fig. 3c). The subgroup correlation for DDIR-positive and DDIR-negative tumours is shown in Supplementary Fig. 8. For signatures reported to predict resistance to ICB, there was a strong correlation with those predicting increased angiogenesis and EMT signalling. Therefore, DDIR-negative tumours with strong angiogenic or EMT signalling were associated with poor responses to NACT.

### Immune responses are induced by DNA-damaging chemotherapy

**Fig. 5:**
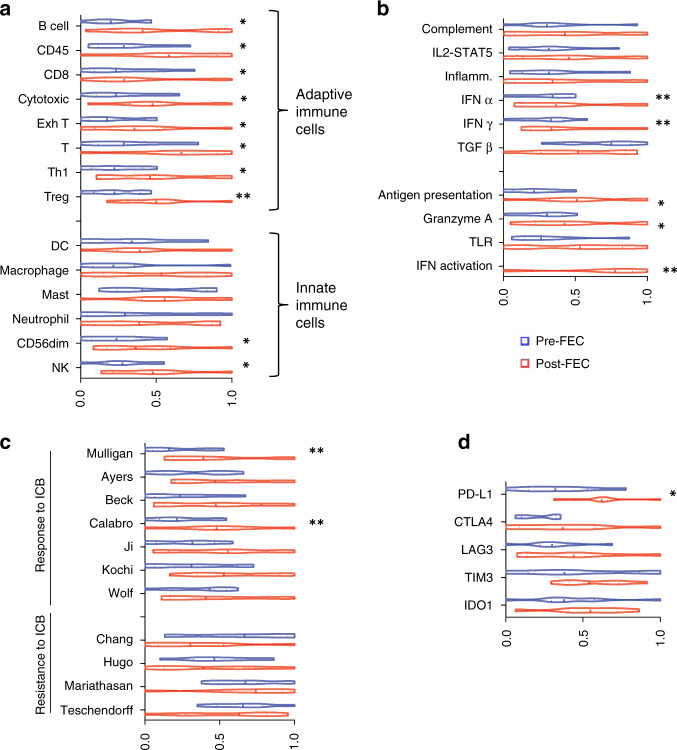
Immune gene expression pre- and following three cycles FEC NACT in DDIR-negative tumours (eight paired samples). **a** Predicted immune cell populations in baseline tumour biopsies and post-FEC in DDIR-negative tumours where blue indicates gene expression score pre-treatment and red following three cycles of FEC chemotherapy. *P < 0.05, **P < 0.01. **b** Upregulation of immune signalling pathways post-FEC in DDIR-negative tumours. *P < 0.05, **P < 0.01. **c** Signature scores: upper panel: signatures reported to predict response to ICB. Lower panel: signatures reported to predict resistance to ICB and an inflamed microenvironment are shown in the lower panel. *P < 0.05, **P < 0.01. **d** Gene expression of immune checkpoints at baseline and following three cycles FEC chemotherapy in DDIR negative tumours. *P < 0.05, **P < 0.01.

Biopsy samples were obtained after three cycles of FEC chemotherapy in a subset of 32 patients. Of these, 12 contained inadequate tumour (<20% tumour content) for RNA extraction. A further three samples failed QC following RNA extraction, leaving 17 samples suitable for subsequent gene expression profiling. Of these, 15 had an RCB 2–3 response to NACT: 8 DDIR-negative tumours and 7 DDIR positive. As expected, tumour content was low in post-FEC samples from tumours that responded to NACT (RCB 0–1). Immune cell populations were analysed as before in baseline and post-FEC-treated samples. DDIR-negative tumours demonstrated an increase in infiltration of adaptive immune-response cells (Fig. 5a, *P* = 0.00246), although there was no notable increase in gene expression signatures identifying innate immune populations (Fig. 5a*, P* = 0.168). A marked increase in immune biologies, in particularly increased interferon α and γ, and interferon activation was identified (*P* < 0.001), with a trend towards reduced TGF-β signalling following NACT (Fig. 5b, *P* = 0.090). Gene expression signatures predicting response to ICB also significantly increased following three cycles of DNA-damaging chemotherapy in these tumours (Fig. 5c*, P* < 0.001). Importantly, in DDIR-negative tumours, there was increased immune-checkpoint gene expression following FEC, although this increase only reached statistical significance for PD-L1 (Fig. 5d). No significant changes were seen in immune gene expression before and after FEC treatment in DDIR-positive tumours (Supplementary Fig. 9).

### Defining characteristics of DDIR-positive responders and non-responders to NACT

No significant differences in clinicopathological characteristics between DDIR-positive tumours with an RCB 0–1 response compared to those with RCB 2–3 were identified, although this did approach significance for breast cancer subtype (*P* = 0.051) (Supplementary Table 3, *P* values in table). Comparison of immune-response signatures in DDIR-positive non-responders (RCB 2–3) and responders (RCB 0–1) did not identify any differences in signature scores (Fig. 3a). There were also no significant differences between angiogenesis and EMT signature scores in DDIR assay-positive non-responders and responders (Fig. 3b; *P* values in Supplementary Table 4, 4A, B).

To address why some tumours with an active immune response at baseline and predicted DNA-repair deficiency did not respond to NACT, we analysed gene expression signatures associated with genomic instability, proliferation, and cell death, comparing non-responders to responders (Supplementary Fig. 10). There was a trend to higher proliferation and cell death signature scores in responding tumours. Signatures associated with genomic instability, including aneuploidy and chromosomal instability, were also higher in responding tumours. However, no clear resistance mechanisms to NACT in these non-responding immune-active tumours were identified.

## Discussion

As outlined above, similar cytotoxic chemotherapy regimens are used to treat breast cancer, regardless of molecular subtype. Despite the evidence that tumours with a DNA-repair pathway deficiency are likely to respond better to DNA-damaging agents (such as anthracyclines), there is no good biomarker in clinical use to predict response to treatment. The DDIR signature was developed using microarray data from *BRCA1/2-*deficient, DNA-repair-deficient tumours with the specific aim of identifying such tumours. Here, we have demonstrated that the 44-gene DDIR signature can predict response to neoadjuvant chemotherapy in unselected early breast cancer patients, across molecular subtypes. Although germline *BRCA1/2* mutation status is unknown in this cohort, the design of the signature means that any *BRCA*-associated cancers will likely be identified as DDIR positive due to their intrinsic DNA-repair deficiency. Furthermore, this feasibility study has clearly demonstrated that the assay can be incorporated into clinical workflows in a timely manner, allowing its adoption into practice.

In recent years, there has been a suggestion that not all breast cancer patients require anthracycline treatment, although there remains a paucity of biomarkers to identify patients likely to benefit from these agents [40]. The findings from this study, taken together with the previous clinical and preclinical studies, suggest that there may be a subgroup of tumours with high sensitivity to anthracycline-containing chemotherapy where anthracycline-sparing regimens should not be considered. Conversely, the signature may also have utility in identifying those patients unlikely to benefit from anthracycline-based chemotherapy (such as FEC or AC), such that these could be omitted to minimise cardiotoxicity and the risk of haematological malignancy. Validation of this hypothesis would however require a direct comparison of anthracycline versus non-anthracycline regimens by DDIR status.

Other studies have reported associations between DNA-repair deficiency and immune infiltration [41–43]. In line with this, the DDIR signature identifies a molecular subgroup of tumours characterised by upregulated immune signalling. In keeping with this, we have shown that DDIR-positive tumours display increased immune infiltration, including both adaptive and innate immune cell populations, compared with DDIR negative tumours. This is consistent with our preclinical data showing that DNA-repair deficiency leads to the presence of cytosolic double-stranded DNA, which is sensed by cGAS, resulting in STING activation and a type I interferon-like response [10]. Importantly, as supported in this clinical study, activation of this pathway is also associated with upregulation of immune-checkpoint gene expression including *PD-L1, CTLA4* and *IDO1* [10, 21].

In this study, we have shown a correlation between DDIR score and TILs, in keeping with previous data which has shown higher DDIR scores to be associated with higher TIL density in a cohort of TNBC patients [9]. TILs have previously been shown to be a prognostic factor in TNBC treated with adjuvant chemotherapy [44]. Furthermore, TILs have been shown to predict pCR in the neoadjuvant setting, albeit with longer-term prognostic value only in HER2 + and TN subtypes [45]. Although we did see higher TIL levels in patients with RCB 0-1 disease compared with RCB 2-3, this finding, unlike the predictive value of DDIR score, did not reach significance. Given that DDIR and TILs have previously been shown to be moderately correlated, it is possible that the ability of TILs to predict pCR in this series is not evident due to the relatively small number of patients included in the series and the fact that they represent all molecular subtypes of disease including luminal cancers. However, the fact that in a small series the DDIR assay is predictive of pCR suggests that this signature provides an objective measure of the biological processes underlying the presence of TILs and thus may have clinical utility across molecular subtypes in breast cancer.

Taken together, this suggests that DNA-repair deficiency/DDIR positivity results in an immune infiltrate which is functionally restricted via upregulation of immune checkpoints. Nonetheless, in this study, DDIR-positive tumours were more likely than DDIR negative tumours to respond to neoadjuvant treatment with regimens containing DNA-damaging agents, presumably due to an intrinsic DNA-repair pathway defect. Importantly, we and others have shown that DNA-damaging agents also induce cytosolic dsDNA and cGAS-STING activation in DNA-repair proficient models [10, 41, 46]. Indeed, we recently carried out an in vitro screen of multiple chemotherapeutic agents for their ability to induce cytosolic dsDNA, identifying anthracyclines as potent cGAS-STING activators [21]. In keeping with this, anthracyclines also induced immune-checkpoint gene expression, suggesting that anthracyclines may be considered as the preferred combination chemotherapy for ICB. The TONIC trial previously confirmed these preclinical findings in the setting of metastatic TNBC, where induction with low-dose doxorubicin was identified as superior to other DNA-damaging agents in promoting responses to ICB [19]. In our study, we have shown that in a small number of immune “cold” tumours (DDIR-negative, *n* = 8), FEC treatment led to increased TILs (albeit a non-significant increase), with significant enrichment of adaptive immune cell populations identified by immune-related gene signatures. Although this represents a small sample, taken in conjunction with other data, this leads us to hypothesise that treatment with chemotherapy regimens containing anthracyclines may result in activation of the cGAS-STING pathway, resulting in higher expression of signatures of the immune response, immune-checkpoint gene expression and immune infiltrates in DDIR negative, immune cold tumours. However, presumably due to an intact DNA-repair pathway, these tumours are less susceptible to DNA-damaging agents and therefore are less likely to exhibit a pCR in this context, unlike DDIR-positive tumours, where there is an innate DNA-repair pathway deficiency as identified by the signature (which was developed from patients and tumours with an innate repair pathway deficiency. The transcriptomic findings from the current study, taken together with data from the TONIC trial, suggest that immune induction followed by ICB may be an effective treatment strategy in immune restricted tumours. In contrast to TONIC, which assessed low-dose therapy for immune induction in the context of pre-treated metastatic triple-negative disease, our study evaluated therapeutic doses of anthracycline-containing regimens in treatment-naïve early breast cancer across molecular subtypes. Further work is required to define the optimal class, dose, and scheduling of induction agents to activate the cGAS-STING pathway by this mechanism and thus to achieve optimal responses to ICB.

There are several studies published evaluating the role of immune-checkpoint blockade in the neoadjuvant setting in early breast cancer, all in triple-negative disease. Both the IMPASSION-031 and KEYNOTE-522 studies combined ICB (atezolizumab and pembrolizumab respectively) with a backbone of anthracycline-taxane chemotherapy and demonstrated an increase in pCR rates with chemotherapy plus ICB when compared with chemotherapy alone (58% vs 41% in IMPASSION-031, and 65% vs 51% in KEYNOTE-522 [47, 48].

Taxanes alone, however, do not cause immunogenic cell death or T-cell activation [49]. While cGAS promotes apoptosis following taxane treatment, cGAS activation in this context does not result in an interferon response [50]. Indeed, our preclinical modelling suggests that taxanes, given in combination with anthracyclines, may suppress cGAS-STING activation via blocking the formation of anthracycline-induced cGAS-activating micronuclei [21]. In keeping with this, in our study, some tumours which initially had an increase in TILs following FEC did not maintain this through taxane treatment, with a subsequent fall in TILs seen at the time of resection, albeit this was only a trend, and did not reach statistical significance. Interestingly, in this context, the NeoTRIP trial in the neoadjuvant setting in early TNBC evaluated nab-paclitaxel in combination with carboplatin + /− atezoluzimab (and did not have an anthracycline component to the chemotherapy backbone) and showed no significant improvement in pCR rates for the ICB arm (43.5% vs 40.8%) [51].

Taken together, our data and that from the clinical trials discussed above suggests the possibility that immune priming may be more effective using anthracycline-based therapy rather than taxanes as a combination strategy, and this is further supported by recently published data [52]. Furthermore, given that the DDIR signature identifies patients with upregulation of immune checkpointing genes, it is possible that this biomarker may be able to identify patients who will respond to ICB. Given the limitations of PD-L1 staining as a biomarker with clinical utility, there is a clear unmet need for a validated biomarker to predict response to immuno-oncological therapeutic strategies. Patients in this study did not receive any treatment with ICB; however, in light of the DDIR signature’s potential to act as a biomarker in this context, further validation in a cohort of patients treated with both neoadjuvant chemotherapy and ICB is merited.

Furthermore, it has also been suggested that in *BRCA1-*deficient TNBC, PARP inhibitors may induce CD8^+^ T-cell infiltration through the cGAS/STING pathway [53]. Consequently, the DDIR signature may also identify a group of tumours with a DNA-repair pathway defect that might benefit from the use of PARP inhibitors or PARPi/ICB combinations.

When considering the DNA-repair proficient, DDIR-negative tumours in this study, we identified increased mast cells, with associated increased activation of angiogenesis, which may contribute to the immune restricted TME observed around these tumours. We also identified increased TGF-β signalling in these tumours at baseline, which was maintained following FEC chemotherapy. TGF-β is proposed to prevent an effective response to ICB by actively excluding lymphocytes from the TME and promoting T-cell exhaustion [27, 54]. This may suggest that therapies targeting this pathway may enhance response to ICB or chemotherapy in these patients. In keeping with increased TGF-β signalling, gene signatures identifying an EMT phenotype were higher in DDIR negative tumours. EMT signalling is associated with the development of stem-cell-like features and associated chemotherapy resistance in breast cancer [55]. Although EMT-targeting therapies in combination with chemotherapy may have clinical utility [56], a key area of interest is the potential of these agents, in particular TGF-β and focal adhesion kinase (FAK) inhibitors, to synergise with ICB [27, 54, 57].

In conclusion, our study validates the DDIR biomarker as predicting response to neoadjuvant chemotherapy in early breast cancer. We have demonstrated the feasibility of utilising the DDIR signature in clinical practice in a timely manner, consistent with its use to make treatment decisions, meriting further large-scale prospective study. In addition, in a small number of cases, we report the induction of immune responses in DDIR negative, immune “cold” breast tumours. Moreover, whilst approval of ICB therapy in the clinical setting has thus far been limited to advanced TNBC, we also observed FEC-induced immune signalling in ER-positive and HER2-positive tumours. This suggests that the combination of anthracycline-based chemotherapy together with ICB in the treatment of early breast cancer may be a rational treatment strategy. Finally, additional validation is required to determine the ability of the signature to predict response to immune-checkpoint therapy in breast cancer.

## Supplementary information

Supplementary Table 1

Supplementary Table 2

Supplementary Table 3

Supplementary Table 4

Supplementary Figures

Supplementary material

## Data Availability

The microarray and sequencing data are openly available in Gene Expression Omnibus with the following accession numbers: Microarray data: GSE180295. RNA-seq data: GSE180280.
