# Supplementary material for: Activation of a cGAS-STING-mediated immune response predicts response to neoadjuvant chemotherapy in early breast cancer
Source: Br J Cancer. 2021 Nov 2;126(2):247–58. doi: 10.1038/s41416-021-01599-0 (PMC8770594; doi:10.1038/s41416-021-01599-0)
Supplement: Supplementary file 1 — Supplementary Table 1 [file 41416_2021_1599_MOESM1_ESM.docx]

**Supplementary Table 1. Optimal retrieval methods and sequential staining steps for Opal multiplex panel 1 and panel 2.**

| **Opal Multiplex Immunofluorescence – Leica Bond Rx** | | | | | | | | | |
| --- | --- | --- | --- | --- | --- | --- | --- | --- | --- |
| **Multiplex Panel** | **Step** | **Biomarker** | **Clone** | **Source** | **Antigen Retrieval** | **Dilution** | **Incubation** | **Detection chemistry** | **Incubation** |
| 1 | 1 | PD-L1 | EIL3N | Cell Signaling | ER2 20 minutes @ 95°C | 1:200 | 30 minutes | Opal 480 @ 1:150 | 30 minutes |
| 1 | 2 | FOXP3 | 236A/E7 | Abcam | ER1 20 minutes @ 95°C | 1:100 | 30 minutes | Opal 620 @ 1:150 | 30 minutes |
| 1 | 3 | CD8 | C8-144B | Dako | ER1 20 minutes @ 95°C | 1:800 | 30 minutes | Opal 690 @1:150 | 30 minutes |
| 1 | 4 | B-Catenin | B-Catenin-1 | Dako | ER1 20 minutes @ 95°C | 1:400 | 30 minutes | Opal 570 @1:150 | 30 minutes |
| 1 | 5 | CD4 | 4B12 | Novocastra | ER1 20 minutes @ 95°C | 1:100 | 30 minutes | Opal 520 @ 1:150 | 30 minutes |
| 1 | 6 | CK | AE1/AE3 | Dako | ER1 20 minutes @ 95°C | 1:100 | 30 minutes | TSA-DIG @ 1:100 + Opal 780 @ 1:25 | 10 minutes  +  60 minutes |
| 1 | 7 | DAPI | n/a | Perkin Elmer | ER2 20minutes @ 95°C | 1:500 | 10 minutes | n/a | n/a |
| 2 | 1 | PD-L1 | SP263 | Ventana | ER2 20 minutes @ 95°C | 1:2 | 30 minutes | Opal 520 @ 1:150 | 30 minutes |
| 2 | 2 | CD68 | 514H12 | Leica | ER2 20 minutes @ 95°C | 1:200 | 30 minutes | Opal 570 @ 1:150 | 30 minutes |
| 2 | 3 | PD-L1 | SP142 | Abcam | ER2 20 minutes @ 95°C | 1:100 | 30 minutes | Opal 620 @ 1:150 | 30 minutes |
| 2 | 4 | CK | AE1/AE3 | Dako | ER2 20 minutes @ 95°C | 1:100 | 30 minutes | Opal 620 @ 1:150 | 30 minutes |
| 2 | 5 | DAPI | n/a | Perkin Elmer | ER2 20minutes @ 95°C | 1:500 | 10 minutes | n/a | n/a |
