# Supplementary material for: Activation of a cGAS-STING-mediated immune response predicts response to neoadjuvant chemotherapy in early breast cancer
Source: Br J Cancer. 2021 Nov 2;126(2):247–58. doi: 10.1038/s41416-021-01599-0 (PMC8770594; doi:10.1038/s41416-021-01599-0)
Supplement: Supplementary file 2 — Supplementary Table 2 [file 41416_2021_1599_MOESM2_ESM.docx]

**Supplementary Table 2:** **Multivariate analysis of predictors of pathological complete response**

|  | **Non-responders**  **(RCB 2-3)**  **n = 28** | **Responders**  **(RCB 0-1)**  **n = 18** | **Multivariable p value** | **Odds ratio (95% C.I.)** |
| --- | --- | --- | --- | --- |
| DDIR  Positive  Negative | 12  16 | 14  4 | 0.0426* | 0.144  (0.02-0.81) |
| TILs  ≤10%  >10% | 19  9 | 9  9 | 0.1251 | 0.24  (0.03-1.36) |
| Grade  2  2/3, 3 | 10  18 | 4  14 | 0.827 | 0.815  (0.11-5.23) |
| ER  Positive  Negative | 18  10 | 9  9 | 0.646 | 0.67  (0.12-3.71) |
| HER2  Positive  Negative | 6  22 | 10  8 | 0.0069* | 10.84  (2.22-76.50) |
| Nodal status  Negative  Positive | 10  18 | 7  11 | 0.978 | 0.98  (0.16–5.87) |
| T stage  T1/2  T3 | 16  12 | 11  7 | 0.7211 | 0.76  (0.15-3.64) |

Multivariate analysis of predictors of pathological complete response, * = p<0.05.
