# Supplementary material for: Activation of a cGAS-STING-mediated immune response predicts response to neoadjuvant chemotherapy in early breast cancer
Source: Br J Cancer. 2021 Nov 2;126(2):247–58. doi: 10.1038/s41416-021-01599-0 (PMC8770594; doi:10.1038/s41416-021-01599-0)
Supplement: Supplementary file 3 — Supplementary Table 3 [file 41416_2021_1599_MOESM3_ESM.docx]

**Supplementary Table 3: Clinico-pathological characteristics in DDIR positive responding (RCB0-1, n=14) versus non-responding (RCB2-3, n=12) tumours**

|  | **RCB0-1** | **RCB2-3** | **P value** |
| --- | --- | --- | --- |
| **Grade:**  2  2/3  3 | 2  2  10 | 2  1  9 | p=0.88^1^ |
| **T stage:**  T1  T2  T3 | 0  8  6 | 1  8  3 | p=0.39^1^ |
| **N stage:**  N0  N1  N2 | 5  6  3 | 5  6  1 | p=0.85^1^ |
| **Subtype:**  ER+ HER2-ve  HER2 +ve  TNBC | 2  7  5 | 7  2  3 | p=0.051^1^ |

1. Chi-squared test, p> 0.05 not significant.
