# Supplementary material for: Activation of a cGAS-STING-mediated immune response predicts response to neoadjuvant chemotherapy in early breast cancer
Source: Br J Cancer. 2021 Nov 2;126(2):247–58. doi: 10.1038/s41416-021-01599-0 (PMC8770594; doi:10.1038/s41416-021-01599-0)
Supplement: Supplementary file 4 — Supplementary Table 4 [file 41416_2021_1599_MOESM4_ESM.docx]

**Supplementary Table 4A: p value results for comparison of immune gene signatures in DDIR positive non-responders (RCB 2-3) v. DDIR responders (RCB 0-1)**

| Gene signature | p value |
| --- | --- |
| CD45 | 0.185 |
| B | 0.934 |
| T | 0.734 |
| CD8 | 0.586 |
| Cytotoxic | 0.420 |
| Exhausted | 0.307 |
| Th1 | 0.813 |
| Treg | 0.413 |
| Dendritic Cells | 0.241 |
| Macrophages | 0.873 |
| Neutrophils | 0.031 |
| NK CD56-dim | 0.197 |
| NK cells | 0.802 |
| Mast cells | 0.969 |
| M2 | 0.054 |
| Complement | 0.423 |
| IL2-STAT5 | 0.250 |
| Inflammation | 0.189 |
| IFN alpha | 0.298 |
| IFN gamma | 0.934 |
| TGF beta | 0.123 |
| PD-L1 | 0.054 |
| CTLA4 | 0.469 |
| LAG3 | 0.309 |
| TIM3 | 0.258 |
| IDO1 | 0.025 |

**Supplementary Table 4B: p value results for comparison of gene expression signatures in DDIR positive non-responders (RCB 2-3) v. DDIR responders (RCB 0-1)**

|  | Gene signature | p value |
| --- | --- | --- |
| Response to ICB | DDIR | 0.698 |
|  | Ayers | 0.516 |
|  | Beck | 0.854 |
|  | Calabro | 0.690 |
|  | Ji | 0.911 |
|  | Kochi | 0.656 |
|  | Wolf | 0.444 |
| Resistance to ICB | Chang | 0.200 |
|  | Hugo | 0.214 |
|  | Mariathasan | 0.118 |
|  | Teschendorff | 0.276 |
| Angiogenesis | Gourley | 0.674 |
|  | Anders | 0.078 |
|  | Bentink | 0.154 |
|  | Mendiola | 0.714 |
| EMT | Knight | 0.495 |
|  | MSigDB EMT | 0.239 |
|  | Lee | 0.682 |
|  | Rokavec | 0.282 |
|  | Wagle | 0.281 |
