# Supplementary material for: Activation of a cGAS-STING-mediated immune response predicts response to neoadjuvant chemotherapy in early breast cancer
Source: Br J Cancer. 2021 Nov 2;126(2):247–58. doi: 10.1038/s41416-021-01599-0 (PMC8770594; doi:10.1038/s41416-021-01599-0)
Supplement: Supplementary file 5 — Supplementary Figures [file 41416_2021_1599_MOESM5_ESM.pdf]

Supplementary Figure 1

A

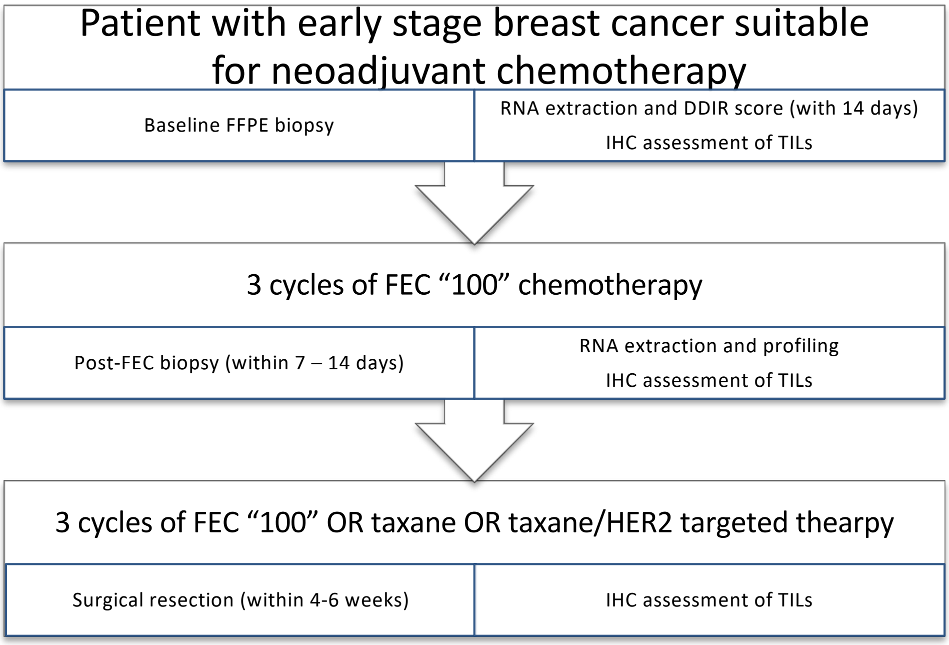

Inclusion criteria:

- Age ≥ 18-years
- WHO performance status 0–1
- T1-3, N0-2 or inflammatory breast cancer
- Adequate left ventricular ejection fraction
- Adequate organ function

Exclusion criteria:

- Pregnancy
- Breastfeeding
- Bilateral breast cancer
- Metastatic disease
- Inability to give informed consent

B

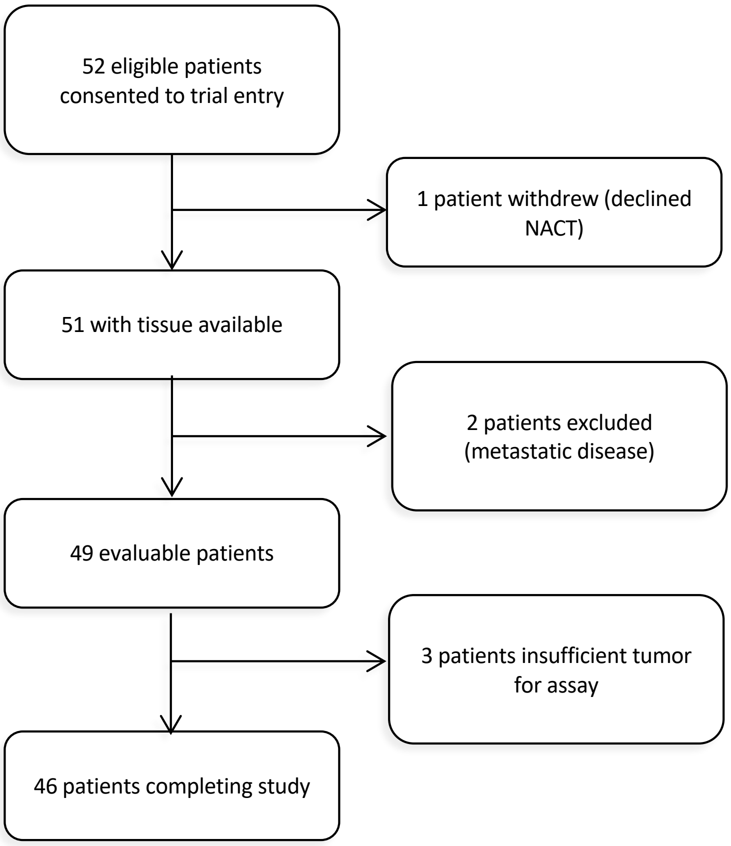

Supplementary Figure 1:
(a) Neo-DDRD trial schema and study inclusion/exclusion criteria.
(b) Recruitment to NeoDDIR clinical trial. CONSORT flow diagram outlining trial recruitment. NACT: NeoAdjuvant ChemoTherapy.

Supplementary Figure 2

a)

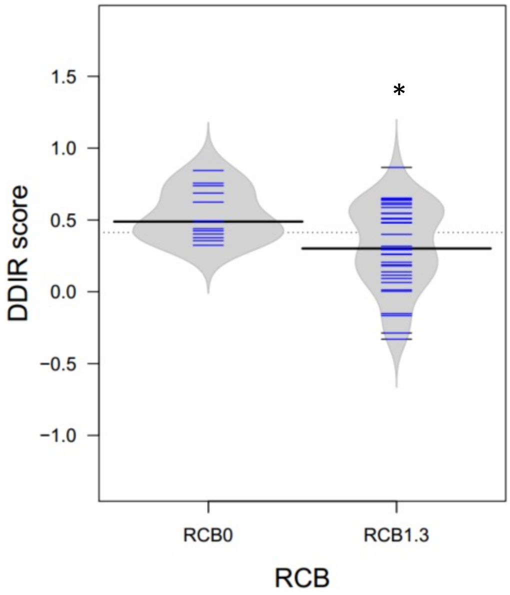

Supplementary Figure 2: DDIR score according to pathological response to treatment

Tumours with a pCR (RCB0) had significantly higher DDIR scores that those with any residual disease (RCB1-3) (p=0.0152, unpaired t-test, 46 evaluable cases)

Supplementary Figure 3

a)

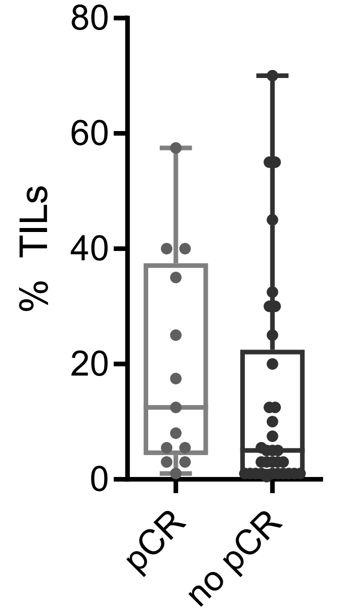

b)

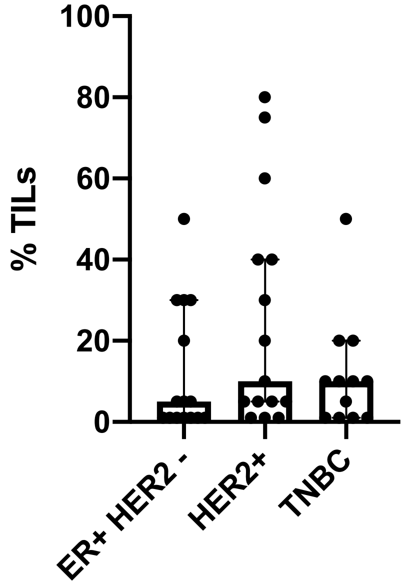

Supplementary Figure 3: TIL levels by pCR and tumour subtype

(a) No significant difference was seen in TILs at baseline in patients with a pCR, compared with patients with any residual disease (p=0.3453, unpaired t-test, 46 evaluable cases)

(b) TILs distribution according to tumour subtype (p=0.0303, one-way ANOVA, 46 evaluable cases)

Supplementary Figure 4

a)

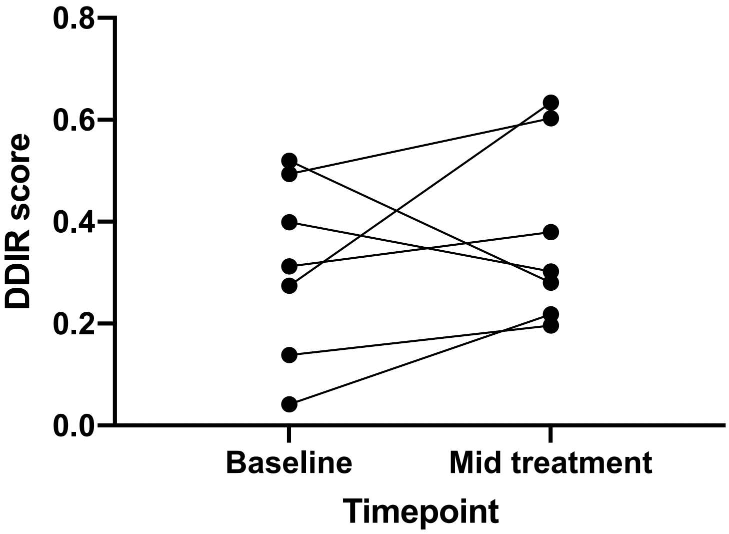

b)

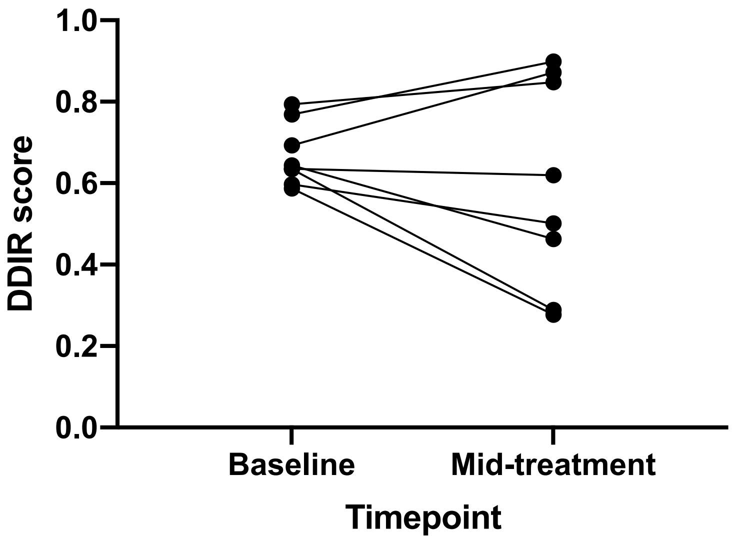

Supplementary Figure 4: Changes in DDIR score following FEC treatment

(a) DDIR score at baseline and after treatment with 3 cycles of FEC-100 in DDIR negative patients (increase in mean DDIR score from 0.31 to 0.38, p=0.52, Mann-Whitney test).

(b) DDIR score at baseline and after treatment with 3 cycles of FEC-100 in DDIR positive patients (decrease in mean DDIR score from 0.64 to 0.56, p=0.57, Mann-Whitney test).

Supplementary Figure 5

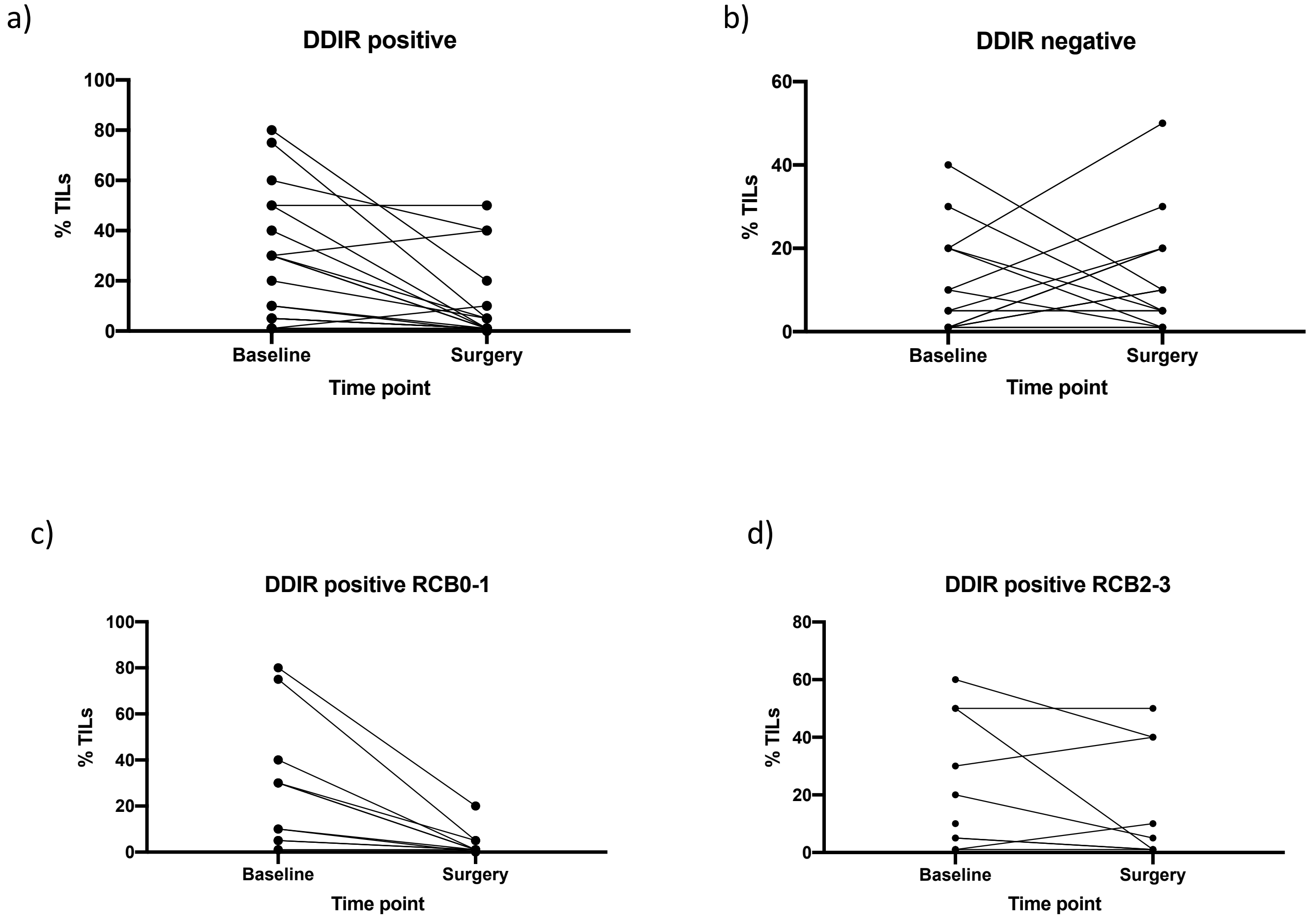

Supplementary Figure 5: TIL changes during chemotherapy treatment

(a) TIL changes pre- to-post-treatment in DDIR positive tumours (21 paired samples, unpaired t test, p=0.0254)

(b) TIL changes pre- to post-treatment in DDIR negative tumours (16 paired samples, unpaired t test, p=0.4431)

(c) TIL changes pre- to post-treatment in DDIR positive tumours with RCB0-1 response to chemotherapy (12 paired samples, unpaired t test, p=0.007)

(d) TIL changes pre- to post-treatment in DDIR positive tumours with RCB2-3 response to chemotherapy (9 paired samples, unpaired t test, p=0.7604)

Supplementary Figure 6

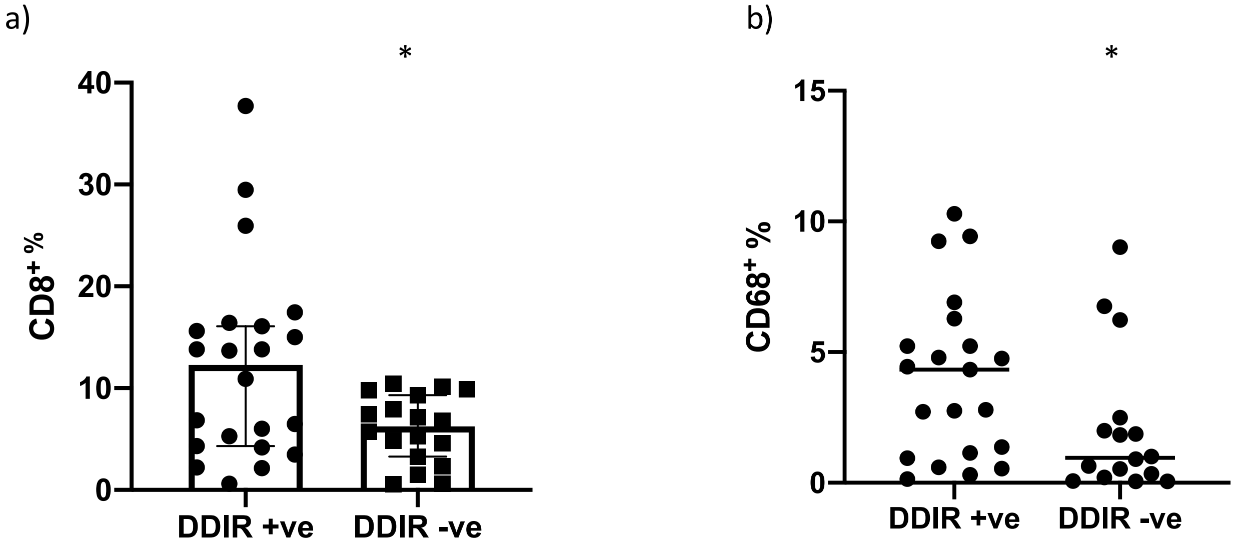

Supplementary Figure 6: Quantification of CD8⁺ and CD68⁺ using multiplex immunofluorescence

(a) CD8 positivity (%) in DDIR positive and negative tumours (median CD8⁺ 12.3% vs 6.24%, p=0.037, Mann-Whitney test).

(b) CD68 positivity (%) in DDIR positive and negative tumours (median CD68⁺ 4.33% vs 0.955%, p=0.029, Mann-Whitney test).

Supplementary Figure 7

a)

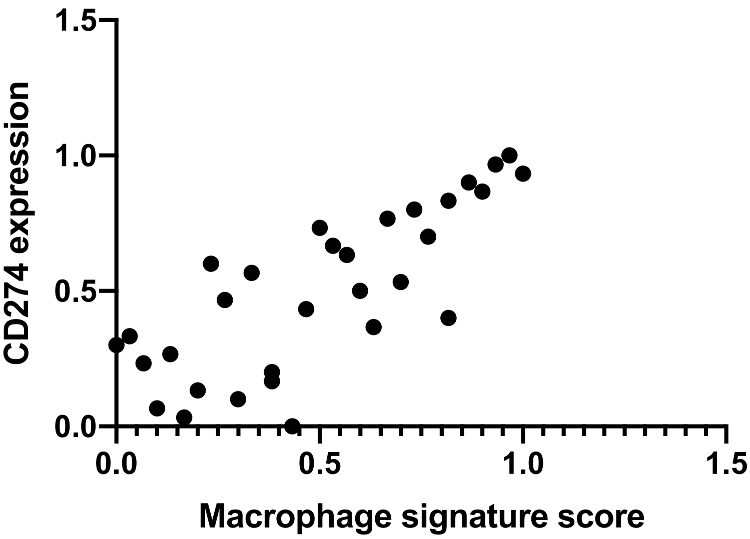

b)

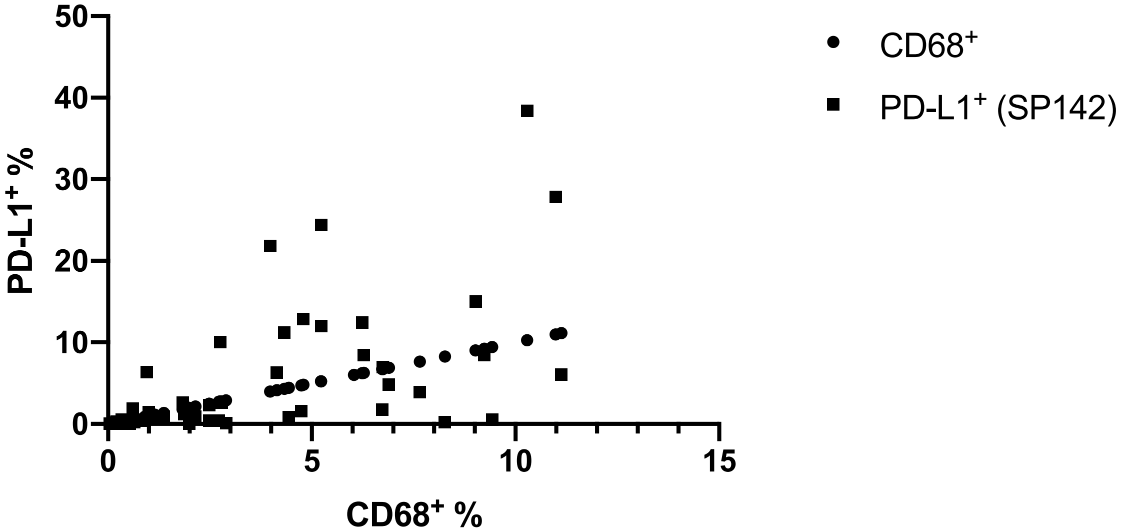

Supplementary Figure 7: Correlation of *CD274* expression and macrophage signature scores, and correlation of CD68/PD-L1 expression

(a) Correlation of *CD274* (PD-L1) expression and Prat macrophage signature score (23) in baseline tumour core biopsies using RNA sequencing data from 31 samples (Spearman correlation, r=0.7869, p<0.001).

(b) Correlation of $CD68^+$ and PD-$L1^+$ (SP142) using quantified multiplex immunofluorescence data (Spearman correlation r=0.6819, p<0.0001).

Supplementary Figure 8

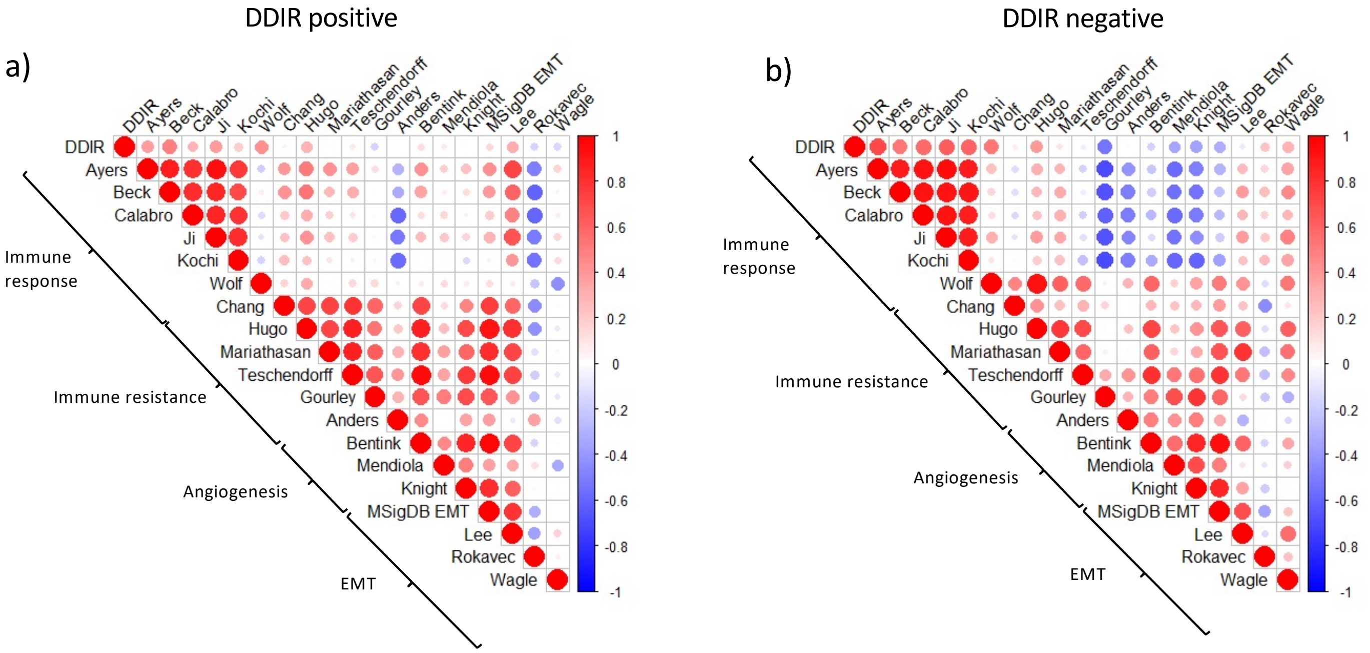

Supplementary Figure 8: Correlations of gene signatures predicting angiogenic, EMT or immune signalling

(a) DDIR positive tumours

(b) DDIR negative tumours

Significance of correlation is shown using size, red indicates positive correlation and blue negative correlation (gene signature references as for Figure 4(b)).

Supplementary Figure 9

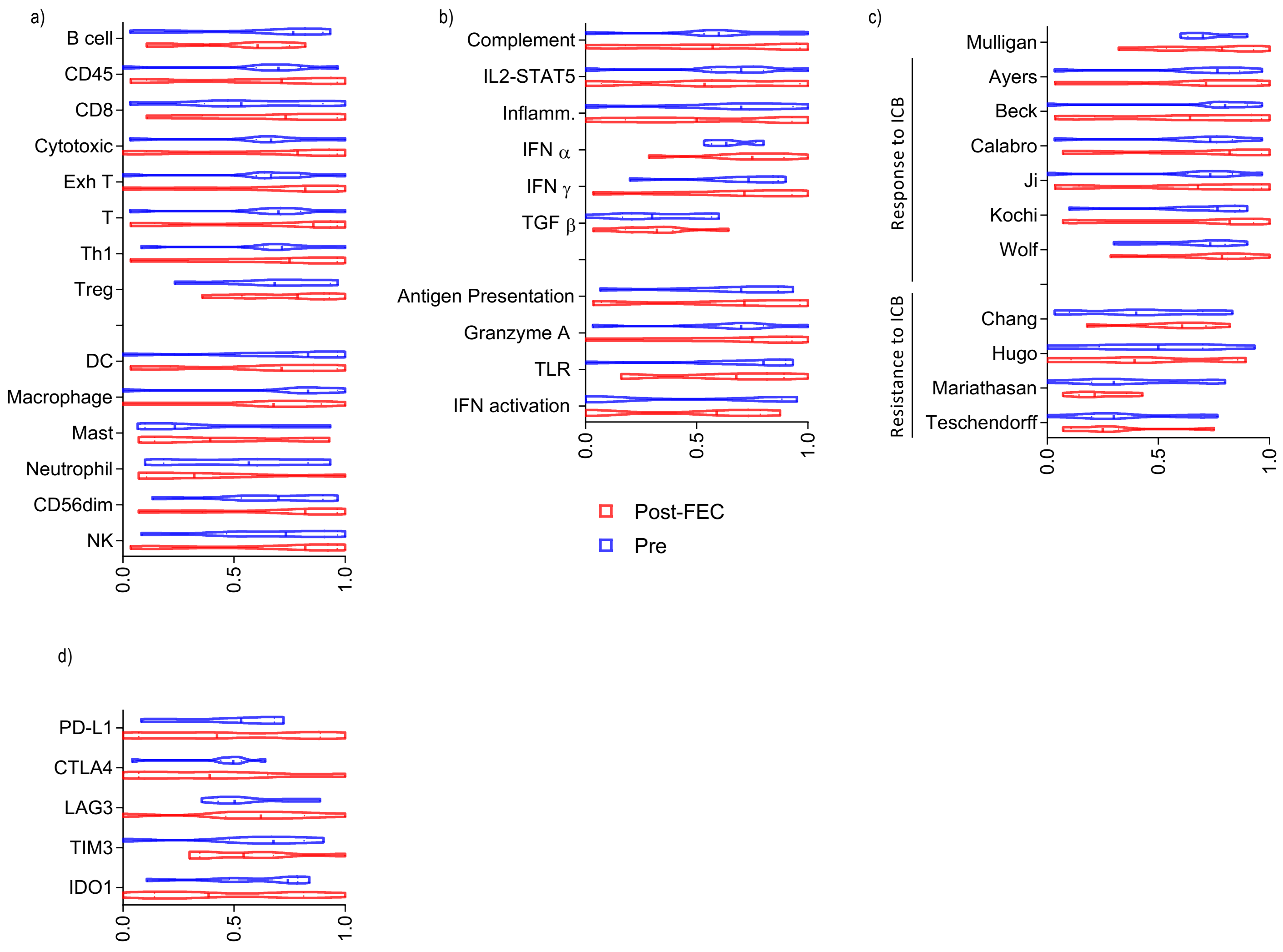

Supplementary Figure 9: Immune gene expression pre- and following 3 cycles FEC NACT in DDIR negative tumours (7 paired samples)

(a) Predicted immune cell populations in baseline tumour biopsies and post-FEC in DDIR positive tumours.

(b) Upregulation of immune signalling pathways post-FEC in DDIR positive tumours.

(c) Signature scores: Upper panel: signatures reported to predict response to ICB ($p<0.001$). Lower panel: signatures reported to predict resistance to ICB and an inflamed microenvironment are shown in the lower panel.

(d) Gene expression of immune checkpoints at baseline and following 3 cycles FEC chemotherapy in DDIR positive tumours.

Supplementary Figure 10

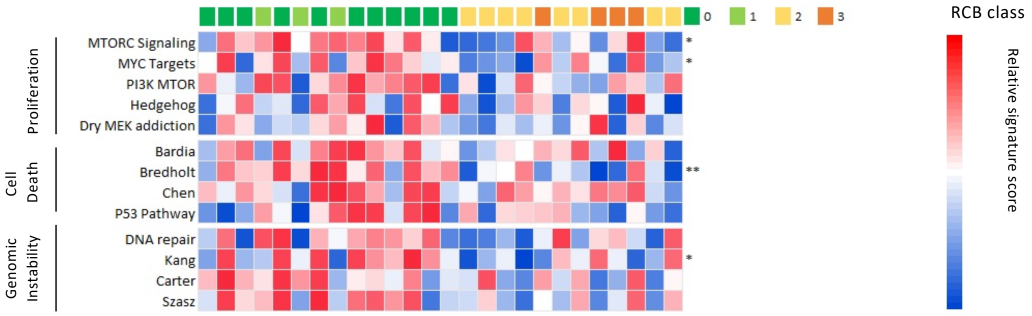

Supplementary Figure 10: Cancer hallmarks in DDIR positive responding (RCB0-1) and non-responding (RCB2-3) tumours
Gene signatures associated with proliferation (panel 1), cell death signalling (panel 2) and genomic instability (panel 3) in DDIR positive non-responders (left) and responders (right) (RCB scores as indicated).
