# Supplementary material for: Activation of a cGAS-STING-mediated immune response predicts response to neoadjuvant chemotherapy in early breast cancer
Source: Br J Cancer. 2021 Nov 2;126(2):247–58. doi: 10.1038/s41416-021-01599-0 (PMC8770594; doi:10.1038/s41416-021-01599-0)
Supplement: Supplementary file 6 — Supplementary material [file 41416_2021_1599_MOESM6_ESM.docx]

**Supplementary material**

**Biomarker evaluation.**

Biomarker evaluation (ER, PR and HER2) was carried out centrally in one UK NEQAS approved tissue pathology laboratory according to Royal College of Pathologists Guidelines. Allred scores of ≥3 (>10% nuclear staining) was taken as the cutoff for positivity for ER and PR. HER2 assessment was carried out centrally using IHC, which was scored by a semi-quantitative method, to give a score range of 0 to 3+. Scores of 3+ were regarded as unequivocally positive, and 0 or 1+ as negative. Borderline scores (2+) were assessed using HER2 dual-colour dual-hapten brightfield in situ hybridisation (DDISH).

**Statistical analysis**

Graphpad Prism v9.0 was used for statistical analysis. Non-parametric distribution of translational data was confirmed by evaluation of frequency distributions. Fishers exact or chi squared analysis and non-parametric testing were carried out as appropriate. Correlation was carried out using the Spearman test for continuous data. Multiple logistic regression analysis was used for multivariate analysis. All p values are unadjusted.

**Data Preparation**

For alignment and expression calculation:

- **StarAlign**^1^: read alignment was performed using StarAlign to the human reference genome GRCh37/hg19. Only uniquely mapped reads were output for downstream analysis and all other reads discarded. Sequences with >=3 base mismatches (against reference) were also discarded.
- **Burrows-Wheeler Aligner**^2^ **(BWA) and samtools flagstat^3^**: were used to determine read alignment to a ribosomal RNA (rRNA) sequence database.
- **Cufflinks^4^** was used to generate gene expression data, represented as Fragments Per Kilobase per Million fragments mapped (FPKM) for each gene present in the human (GRCh37) ENSEMBL annotation file.  “No-effective-length-correction” was set to true, and RNA Exome libraries are stranded so the “fr-firststrand” flag was specified.
- **HTSeq**^5^**:** was used to count the number of reads aligning to each gene present in the human (GRCh37) ENSEMBL annotation file.

**Data Analysis Methodologies**

Category One: included in the logic to assess sequencing quality and suitability of data for downstream analysis.

- *Mean target coverage (alternative name: SeqQC)* This indicates the mean number of aligned, de-duplicated reads at each base targeted by RNA Exome.  Samples achieving mean target coverage less than 14.24X are deemed to have failed alignment QC and are not recommended for inclusion in downstream analysis.
- *Duplication rate* This is given as the percentage of read pairs mapping to identical locations on the human genome.  High duplication rates could indicate an issue with technical duplicates arising from PCR artefacts.  Samples with duplication rates >60.0% are flagged with a warning and recommendation for further review.

**clara^T^**

Only samples with a mean target coverage > 14.24 were taken through for analysis with the clara^T^ software. clara^T^ Total mRNA Report provides a comprehensive overview of tumour profiles using gene expression signatures and single gene analytes. These representative signatures and genes are categorised by the Hallmarks of Cancer. Content includes Almac proprietary assays and public signatures, all published and biologically relevant in either pre-clinical or clinical sample cohorts.

Signatures within this report have been reviewed and implemented based on information from the publications. Based on limited information, signatures denoted with an (*) have been implemented with some modifications to those described in the relevant publications. For further details on each gene signature please refer to the relevant publications in the Bibliography. Single genes within this report have been selected as analytes relevant to each biology based on the Molecular Signatures Database Hallmark Gene Sets (Liberzon et al. 2015; Copyright ©2004-2017 Broad Institute Inc. and subject to the terms and conditions of the Creative Commons Attribution 4.0 International License). The DDIR signature score for RNAseq data was generated by the clara^T^ software.

**Multiplex Immunofluoresence staining**

The multiplex panels used for immunofluorescence staining were as follows:

Multiplex panel 1 contained biomarkers for the identification of the immune checkpoint protein PD-L1 (clone E1L3N), T regulatory cells (FOXP3 clone 236A/E7), T cells (CD8 clone C8-144B), β-Catenin (clone β-catenin-1), T helper cells (CD4 clone 4B12), epithelial cells (cytokeratin clone AE1/AE3), and all other nucleated cells (DAPI).

Multiplex panel 2 contained: biomarkers for PD-L1 (clones SP263 and SP142), macrophages (CD68 clone 514H1), epithelial cells (cytokeratin clone AE1/AE3) and all other nucleated cells (DAPI).

The multiplex (mIF) stained slides were scanned using an Akoya Vectra Polaris (Akoya Biosciences, Marlborough, MA.). Fluorescence images were imported into the open-source, digital image analysis software, QuPath (version 0.2.3). The captured specimen was annotated using the simple tissue detection method. Each annotation underwent a rigorous quality control process where the quality of the tissue slides were assessed to determine their suitability for analysis. Areas with tissue folds, artefacts, or necrotic cells were removed from the annotations. Quality control was conducted by an image analyst and confirmed by a second reviewer with experience in image analysis. Cell detection was then carried out using the DAPI channel to detect all nucleated cells. Tumour cells were identified using the CK channel at set thresholds based on the staining intensity. The biomarkers CD68, CD8 and PD-L1 (SP142 clone) were identified as present on cells within the tumour or stroma areas, which were then classified as positive or negative for each biomarker based on consensus thresholds,. CD68^+^ cells were identified as macrophages while CD8^+^ cells were identified as T-cells. A composite classifier was created based on the individual thresholds for CD68 and PD-L1 to identify PD-L1^+^/CD68^+^ cells or PD-L1^+^ macrophages. The measurements obtained from the analysis in QuPath were then exported for further analyses.

**Supplementary References**

1.                  Dobin A, Davis CA, Schlesinger F, Drenkow J, Zaleski C, Jha S, et al.  STAR: ultrafast universal RNA-seq aligner.  Bioinformatics 2013; 29: 15-21.

2.                  Li H & Durbin R (2009) Fast and accurate short read alignment with Burrows-Wheeler Transform. Bioin e63243.

3.                  Li H, Handsaker B, Wysoker A, Fennell T, Ruan J, Homer N, et al. (2009) The Sequence alignment/map (SAM) format and SAMtools. Bioinformatics, 25, 2078-2079.

4.                  Trapnell C, Williams BA, Pertea G, Mortazavi A, Kwan G, van Baren MJ, et al.  Transcript assembly and quantification by RNA-Seq reveals unannotated transcripts and isoform switching during cell differentiation.  Nature Biotechnology 2010; 28: 511-515.formatics, 25:1754-60.

5. Anders S, Pyl PT, Huber W. HTSeq – a Python framework to work with high-throughput sequencing data. Bioinformatics 2015;31(2):166-9.
